# Antimicrobial Silver Nanoparticles for Wound Healing Application: Progress and Future Trends

**DOI:** 10.3390/ma12162540

**Published:** 2019-08-09

**Authors:** Federica Paladini, Mauro Pollini

**Affiliations:** Department of Engineering for Innovation, University of Salento, Via Monteroni, 73100 Lecce, Italy

**Keywords:** wound, infection, silver, nanoparticles

## Abstract

Recent data have reported that the burden of infections related to antibiotic-resistant bacteria in the European Union and European Economic Area (EEA) can be estimated as the cumulative burden of tuberculosis, influenza, and human immunodeficiency virus (HIV). In wound management, the control of infections represents a crucial issue and a multi-billion dollar industry worldwide. For diabetic wounds ulcers, in particular, infections are related to the majority of amputations in diabetic patients, which today represent an increasing number of the elderly. The greatest barrier to healing is represented by the biofilm, an organized consortium of bacteria encapsulated in a self-produced extracellular polymeric substance with high resistance to conventional antimicrobial therapies. There is an urgent need for novel anti-biofilm strategies and novel antimicrobial agents and, in this scenario, silver nanotechnology has received tremendous attention in recent years in therapeutically enhanced healthcare. Due to its intrinsic therapeutic properties and the broad-spectrum antimicrobial efficacy, silver nanoparticles have opened new horizons towards novel approaches in the control of infections in wound healing. This review aims at providing the reader with an overview of the most recent progress in silver nanotechnology, with a special focus on the role of silver in the wound healing process.

## 1. The Wound Healing Process

The skin, the largest organ of the body, has a crucial role in sensory functions, homeostasis, control of temperature, and protection against pathogens, toxins, and trauma [1]. The disruption of the integrity of skin determines the formation of a wound, which can occur as part of a disease or can have an accidental or intentional etiology [2,3]. Wounds can arise from surgical intervention, from an injury, or from other factors and conditions, including pressure, shear, diabetes, or vascular diseases. They can be classified as acute wounds (i.e., surgical wounds and burns) and chronic wounds (i.e., leg, diabetic foot (DFUs), and pressure ulcers) [4]. A chronic wound can be defined as a difficult to heal wound/ulcer that has failed to proceed towards anatomic and functional integrity within 3 months, and that has failed to heal through an orderly and timely reparative process [5].

Aimed at restoring tissue integrity and functions, the wound healing process involves multiple cellular and extra-cellular pathways through overlapping phases, namely hemostasis/inflammatory phase, proliferative phase, and remodeling phase [2,3]. In a vascular inflammatory response, the damaged blood vessels contract and coagulation occurs through an aggregation of thrombocytes in a fibrin network. Then, during the proliferative stage, angiogenesis and reepithelialization occur, leading to the healing of the wound. In the remodeling phase, the maximum tensile strength is achieved through reorganization, degradation, resynthesis of the extracellular matrix, and remodeling of the granulation tissue.

Multiple growth factors and cytokines released at the wound site strictly regulate wound healing [6,7,8] and, due to the complex nature of the process, many factors can interfere delaying wound healing, increasing patient morbidity and mortality, and resulting in a low cosmetic outcome and significant discomfort and distress [2,9]. From a macroscopic perspective, wound healing depends on multiple parameters, such as wound size, depth, location, patient age, and the presence of local or systemic disease [10]. Other factors, such as nutritional and immunological status, stress, smoking, diabetes, obesity, and hypertension, can also have an impact on wound healing [11,12], together with a generally increased longevity of the elderly population, which has increased the prevalence of non-healing ulcers [12]. In aged skin, the microcirculation, which is very important in wound healing, shows impaired vasoregulation, changed inflammatory responses, and fewer progenitor cells.

The incidence of chronic wounds is increased among older adults and has a great impact on the quality of life [13]. Whatever the cause, chronic wounds have a significant physical, mental, social, and economic impact on patients and the healthcare system so that the phenomenon of chronic wounds has been called the ‘Silent Epidemic’ [4,14].

In the United States, chronic ulcers affect more than 6 million people, with increasing numbers in the growing elderly and diabetic populations [15]. In the United States, 20 million people are affected by diabetes, and this number is expected to double by the year 2030; 15% of these patients are affected by diabetic foot ulcers, which are related to most cases of amputations [9]. Underlying conditions, such as diabetes and neuropathy, can also have an effect on the nature of the wound fluid, varying considerably during the healing process as a consequence of changes in the microenvironment and tissue remodeling progresses. In the United Kingdom, 650,000 patients are affected by some form of chronic wound, with estimated costs of approximately £3 billion per year [16,17]. Guest et al. analyzed the annual levels of health care costs associated with the management of different wound types by the United Kingdom’s National Health Service (NHS) in 2012/2013. During this period, the cost per unhealed wound ranged from £1719 to £5976 per patient, 135% more than that of a healed wound [18]. The market of wound care products exceeds US$15 billion, with single diabetic ulcer costing nearly US$50,000 and over US$25 billion per year estimated for chronic wounds on the medical system [9]. A significant number of treatments are available for the management of wounds and burns, representing a multi-billion dollar industry worldwide [19]. In the USA alone, the annual expenditure estimated for wound management exceeds one billion dollars [2].

An ideal dressing should mimic the extracellular matrix and should be characterized by biological stability, flexibility, and capability to remove the wound exudate while providing a moist environment at the wound site. It should also protect the wound from external hazards and bacterial infections, should enhance epidermal migration, and promote angiogenesis and connective tissue synthesis [20,21].

Many wound dressings have been developed for protecting the wound from infection and for promoting the wound healing process [9]. Current wound healing approaches involve the use of autografts, allografts, cultured epithelial autografts, and wound dressings based on biocompatible and biodegradable polymers, such as collagen, chitosan, and hyaluronic acid, which have been approved for wound dressing by the Food and Drug Administration (FDA) [22]. More than 3000 products have been designed, including conventional wound dressings, dressings incorporating growth factors and biological molecules, for improved cellular migration and extracellular matrix (ECM) production, and also skin substitutes incorporating patient-derived cells [19,20,21].

Recently, wound dressings with antimicrobial agents have been considered as a valuable chance to control bacterial colonization and infection in wound healing [23].

## 2. Wound Infections

The control of infections represents a crucial aspect in the management of chronic wounds [24]. While bacteria are a normal part of skin flora and wounds, 10^5^ bacteria have been suggested as the critical threshold between colonization and clinically relevant infection [9]. In damaged skin, bacteria can reach the underlying tissues causing inflammation, which leads to the release of proteases and reactive oxygen species from inflammatory cells [10,23].

Increased levels of endotoxins produced by bacteria elevate the levels of proinflammatory cytokines, thus decreasing the production of growth factors and the deposition of collagen in wounds and delaying wound healing [25]. Then, the infection can progress from contamination to colonization and can be complicated by the presence of biofilm [26], an organized consortium of bacteria encapsulated in a self-produced extracellular polymeric substance (EPS) made of polysaccharide, protein, and DNA [27]. Biofilms have been identified in 60% of biopsy specimens from chronic wounds and 6% of biopsy specimens from acute wounds [10]. Within the biofilm, the microorganisms represent the greatest barriers to healing due to their resistance to conventional antimicrobial therapies [28] and to multiple tolerance mechanisms, including both phenotypic and genetic resistance [29]. *Pseudomonas aeruginosa* (*P. aeruginosa*), for example, exhibits multiple resistance mechanisms, such as decreased permeability, expression of efflux systems, production of antibiotic inactivating enzymes, and target modifications [30]; other multi-drug-resistant nosocomial organisms include MRSA, vancomycin-resistant enterococci, and Klebsiella pneumoniae (*K. pneumoniae*) [31].

Improper use of antibiotics and non-rational antibiotic therapies are considered the major causes of this drug resistance [31]. Recent data have reported the estimated burden of infections related to antibiotic-resistant bacteria in the European Union and European Economic Area (EEA) is similar to the cumulative burden of influenza, tuberculosis, and HIV, with a growing trend between 2007 and 2015 and a major onset in hospitals and health-care settings [32]. From European Antimicrobial Resistance Surveillance Network (EARS-Net) data collected in 2015, 671,689 infections were associated with antibiotic-resistant bacteria, with 63.5% associated with health care and 33,110 deaths. The burden has increased since 2007, with the highest incidence in Italy and Greece [32]. Increased risk of hospital infections and widespread emergence of microorganism resistance also determines increased treatment costs, further complicated by potential hypersensitivity reactions to antibiotics [12,31]. Antibiotic treatment alone is often inadequate to overcome biofilm infections, and a multidisciplinary approach involving microbiologists and clinicians is strictly necessary [33].

In chronic wound management, biofilm determines high inflammation due to excessive and prolonged stimulation of nitric oxide, inflammatory cytokines, and free radicals, thus leading to delayed healing [26].

Today biofilm, which is implicated in most non-healing wounds and in wounds infections, has stimulated significant research interest and debate in wound care. [34]. Bessa et al. identified bacterial pathogens in infected wounds and their resistance profile to the most common antibiotics. Three hundred and twelve wound swab samples collected from 213 patients with diverse types of wounds were analyzed, and a total of 28 species were isolated. The most common bacterial species resulted in *Staphylococcus aureus* (*S. aureus*), *Pseudomonas aeruginosa* (*P. aeruginosa*), *Proteus mirabilis, Escherichia coli* (*E. coli*), *and Corynebacterium spp.* In addition, *S. aureus/P. aeruginosa* association was found in polymicrobial infections together with high resistance to the majority of antibiotics shown by Gram-negative bacteria [35]. *Staphylococcus aureus* is commonly isolated from infected wounds both in animals and humans [36]; *Pseudomonas* species are frequently isolated in burn wounds, which are often characterized by the presence of exudate and, hence, by a moist and nutrient-rich environment for bacterial growth [37]. Burns, chronic wounds, post-surgical wounds, and diabetic ulcers have prolonged healing times and, sometimes, even fail [38].

A diabetic wound is often associated with the formation of chronic and refractory ulcers and, due to local and systemic factors, the healing process does not progress towards proliferation and maturation phases, inhibiting the synthesis of various cells, cytokines, proteins, and growth factors and affecting proliferation and migration of fibroblast and keratinocytes [39]. The skin in diabetic patients is also more susceptible to skin infections, and foot infections occurring as a result of skin ulceration represent a serious risk for limb amputation [38,40,41].

A quick diagnosis and immediate treatment are strictly necessary [38], along with the development of wound dressings with the capability to prevent bacteria penetration into the wound and to avoid the growth of microorganisms [37]. In particular, novel anti-biofilm strategies and microbiology research in biofilm infection and antimicrobial resistance are urgently necessary. Moreover, diagnostic methods incorporating routine microbiological procedures and more sophisticated clinical laboratory testing are needed [42,43]. Anti-biofilm research has generated many routes for potential novel therapies. Combining biofilm research with therapeutic development, the treatment of biofilm infections should be significantly increased [44].

Although some progress has been made in the definition of novel anti-biofilm agents, the treatment options are still limited by the high cost and complexity, and there is a growing interest in exploring new options, including the vast repertoire of bioresources [23]. Nanotechnology can provide promising approaches to prevent drug-resistant biofilm infections of medical devices and to develop a new generation of biomedical and industrial applications [45,46]. Due to the intrinsic potential bactericidal and fungicidal properties exhibited by some nanoparticles (NPs), they have been employed in different therapeutic approaches demonstrating efficacy in wound care and related biomedical issues and addressing nanomedicine as a valuable option for developing new antimicrobial agents [37,45].

## 3. Antimicrobial Silver Nanoparticles

Silver and other non-antibiotic treatments were abandoned when penicillin and other antibiotics were discovered but, today, silver has received significant attention because of the emergence of antibiotic-resistant strains and its low tendency to develop resistance [47]. Due to intrinsic therapeutic properties and multi-site action, silver nanoparticles exhibit a broad-spectrum antibacterial capability against many micro-organisms and demonstrate a huge potential to overcome the emerging issues in the area of microbial resistance in different applications [48,49,50] and, in particular, in therapeutically enhanced healthcare. Silver nanoparticles (AgNPs) have demonstrated huge potential in different applications, such as in detection and diagnosis, drug delivery, for coating of biomaterials and devices, for novel antimicrobial agents, and in regeneration materials [51]. In recent years, the antimicrobial feature of AgNPs has led to increased demand for its medical applications [52], including wound dressings, artificial implantation, and antitumor drug carriers [53,54]. Other examples include the use of NPs as a coating for implantable medical devices, for preventing infection and promoting wound healing, in antibiotic delivery, microbial diagnostics, and in antibacterial vaccines to control bacterial infections [55]. The broad-spectrum antimicrobial activity of AgNPs has encouraged the development of many AgNPs-based products for the textile, food, and medical applications [56]. In daily life, AgNPs have been proposed in silver-based systems for air/water filtration, textile materials, animal husbandry, biomedical, food packaging, etc. [57].

Due to the huge potential of AgNPs in biomedical application, many efforts have been made to understand the complex mechanisms of their biological activity, which depends on various physicochemical parameters including dimension, shape, concentration, surface charge, colloidal state, and surface coatings [51,56]. It is accepted that Ag nanoparticles with small diameter have a superior antimicrobial effect than those with a larger diameter, and their antibacterial activity is higher than their bulk equivalents [47]. Below 10 nm, the activity of the silver nanoparticles is mainly attributed to the nanoparticle itself, while in larger particles, it predominantly occurs through the silver ions [58].

The mechanism in different biological systems still remains enigmatic [50], but it has been assessed that, due to the nanometric size and increased surface area, AgNPs can destroy the membrane, cross the body of the microbe and create intracellular damages [46]. Total disintegration of the cells and lipopolysaccharide (LPS) removal occurs through membrane protrusions binding to NPs, which enter the cell by electrostatic attraction. Oxidative stress induction, metal ion release, and non-oxidative mechanisms are also accepted mechanisms for explaining the antimicrobial activity of silver nanoparticles [55]. In contact with bacteria, AgNPs tend to aggregate in correspondence with the bacterial membrane. As described by Le Ouay et al., other mechanisms involve Ag^+^ species, which can inactivate biological systems, such as DNA, peptides, or cofactors recognized as targets by these ions [48]. In a recent study, Wang et al. investigated the molecular mechanisms underlying the interaction between silver and proteins in *E. coli*. By using a homemade liquid chromatography gel electrophoresis inductively coupled plasma mass spectrometry (LC-GE-ICP-MS), the authors were able to map Ag^+^ binding proteins and to demonstrate the capability of Ag ions to interfere with many biological processes in *E. coli*, disrupting the key enzymes involved in glycolysis, in tricarboxylic acid (TCA) cycle and in the oxidative defence systems. The authors also mentioned an initial study on *S. aureus* and concluded that Ag^+^ has different targets between gram-negative and gram-positive bacteria [59]. Kalishwaralal et al. tested biologically synthesized AgNPs on biofilms produced by *Pseudomonas aeruginosa* and *Staphylococcus epidermidis*, demonstrating that silver nanoparticles inhibited both the growth of bacteria and their ability to synthesize exopolysaccharide. As an anti-biofilm mechanism, the authors also indicated the diffusion of AgNPs through the pores for nutrient transportation in the EPS layer [60]. Another mechanism through which metal AgNPs and Ag ions reduce the formation of biofilms is the interruption in quorum sensing (QS) [61], a bacterial gene expression system controlled by small signaling molecules. Ravindran et al. studied the effect of phytosynthesized silver nanoparticles as anti-quorum sensing and anti-biofilm agent against a multidrug-resistant human pathogen, namely *Serratia marcescens*, and they found reduced QS-dependent virulence factors, including EPS production [62]. The research performed by Singh et al. demonstrated that the treatment with silver nanoparticles interrupt the quorum sensing signaling in *Pseudomonas aeruginosa*, also inhibiting several virulence factors and the development of biofilm [63]. As assessed by Satish et al., biofilm formation and EPS production are controlled by quorum sensing also in vibrios. In addition, an important parameter in biofilm formation is its maintenance and architecture. In their studies, the authors demonstrated that AgNPs were effective in adversely affecting the structure of vibrios biofilms and in reducing the colonies [64].

While antibiotic molecules can target only a specific aspect of the bacteria life, silver ions bind non-specifically to many different targets, thus affecting bacteria in many components of their metabolism and structure at the same time [48].

AgNPs have achieved high commercialization, with 55.4% of the total nanomaterial-based consumer products available on the market (313 out of 565 products) [65,66] being AgNPs. Acticoat™ and Bactigras™ (Smith & Nephew), Aquacel™ (ConvaTec), PolyMem Silver™ (Aspen), and Tegaderm™ (3M) are examples of silver-based biocomposites approved by the United States (US) FDA for wound-dressing applications. Promising results have also been reported for AgNPs-incorporated biomaterials, such as modified cotton, bacterial cellulose, chitosan, and sodium alginate [51]. Various routes have been developed to synthesize silver nanoparticles, including physical, chemical, and biological procedures, with an increased interest in eco-friendly processes that do not involve the use of hazardous reagents and minimize the environmental impact [49].

Many silver treatments based on both incorporation and deposition of AgNPs on biomaterials surfaces have been developed through different technologies. For examples, Park et al. developed a technique to incorporate silver nanoparticles onto polyamide thin-film composite membranes via arc plasma deposition (APD) to obtain antibacterial properties and improve membrane performance at the same time [67]. Brobbey et al. simultaneously synthesized and deposited silver NPs onto a glass surface with the liquid flame spray aerosol technique and, then, adopted a hexamethyldisiloxane plasma coating to immobilize the NPs [68]. Silver nanoclusters/silica coatings were deposited by Irfan et al. on cotton fabric for surgical gowns by using radio frequency (RF) co-sputtering, embedding the silver nanoclusters in the silica matrix and controlling silver nanoclusters dimensions and density by manipulating sputtering process parameters. No reducing and/or stabilizing agents were required [69]. Another technique was described by Ibrahim et al. for the development of silver-coated cotton. The authors adopted colloidal silver formulations, where cotton fabric samples were immersed. Then, AgNPs were fixed on the fabric surface using a curing process, utilizing gamma-irradiation or a thermal method [70]. A method to synthesize and deposit silver nanoparticles was developed by Pollini et al. through an in situ photochemical deposition process. The silver coating was characterized by excellent adhesion to the substrate and by long-term broad-spectrum antibacterial efficacy on different natural substrates for biomedical use [71,72,73], such as cotton gauzes and flax, demonstrating good antibacterial and antifungal properties even in contact with simulated infected exudate [74,75].

**Figure 1:**
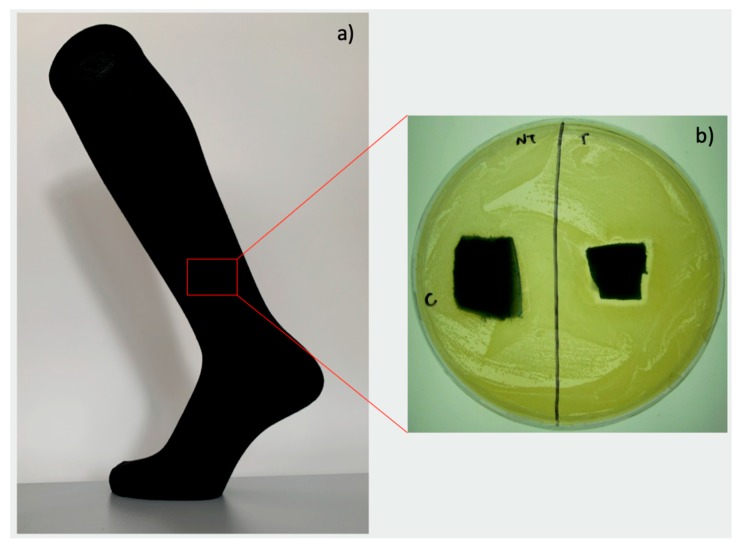
Silver treated device for prevention of infections in diabetic foot disease (**a**); Agar diffusion test demonstrating the good antibacterial properties of the device (**b**).

In Figure 1a, a sock for a diabetic foot is reported, along with the antibacterial tests demonstrating the antibacterial efficacy of the silver treated device. The cotton substrate was treated by Pollini et al. by using a silver-based solution containing a silver precursor and a photo-reducing agent; then, UV irradiation of the substrate provided the photochemical synthesis and deposition of the silver. The silver coating strongly adhered to the textile material and demonstrated durable effectiveness even after several washing cycles. In Figure 1b, the antibacterial capability on *E. coli* tested through agar diffusion tests is reported, and an inhibition area to bacterial growth can be observed in correspondence with the silver treated sample (right) with respect to the untreated sample (left).

Other methodologies, such as cluster beam deposition, have been described in the literature to synthesize Ag nanoparticles, involving physical methods that allow control and deposition of NP without chemical agents on hard substrates [76] or on polyvinyl fibers [77], hence determining their mechanical behavior [78]. In combination with other elements to stabilize the coating, improved performances, and wide bactericidal spectrum have been achieved [79,80], which might have a significant impact on the application of Np in the field.

AgNPs can interact with different microorganisms, and also with mature bacterial biofilms, and therefore, could be used as broad-spectrum antimicrobials [46]. For Example, Kasithevara et al. tested AgNPs against drug-resistant *P. aeruginosa*, *Staphylococcus aureus*, and coagulase negative staphylococci CoNS isolates from post-surgical wound infections, demonstrating the high toxicity of silver against the bacterial strains and a dependence of the efficacy on the concentration of AgNPs [81]. In their experimental studies, Panpaliya et al. demonstrated that silver nanoparticles solution exhibited a higher bacteriostatic and bactericidal effect than chlorhexidine against five oral pathogenic bacteria [82]. Indeed, while the mean minimum inhibitory concentrations (MIC) of Chlorhexidine gluconate was between 150 ± 55.90 μg/mL and 450 ± 111.8 μg/mL, the MIC of AgNPs was significantly lower, ranging from 2.82 ± 0.68 μg/mL to 90 ± 22.36 μg/mL [82]. AgNPs were also successfully used against *Pseudomonas aeruginosa*, which has great intrinsic antimicrobial resistance [83], and against pathogenic microorganisms including *Vibrio parahaemolyticus*, *Salmonella enterica*, *Bacillus anthracis*, *Bacillus cereus*, *Escherichia coli,* and *Candida albicans* [84]. Perez-Diaz et al. showed that AgNPs effectively inhibited the growth of planktonic *Streptococcus mutans (S. mutans)* clinical isolate with a dose and size dependence, and also killed established *S. mutans* biofilms [85]. The average MIC of AgNPs with dimensions of 9.5 nm, 25.9 nm, and 78.7 nm was 4 μg/mL, 8 μg/mL, and 16 μg/mL, respectively. These values were better than that achieved with commercial antibiotic oxacillin, with an average MIC of 16 ± 0 μg/mL [85]. 

**Figure 2:**
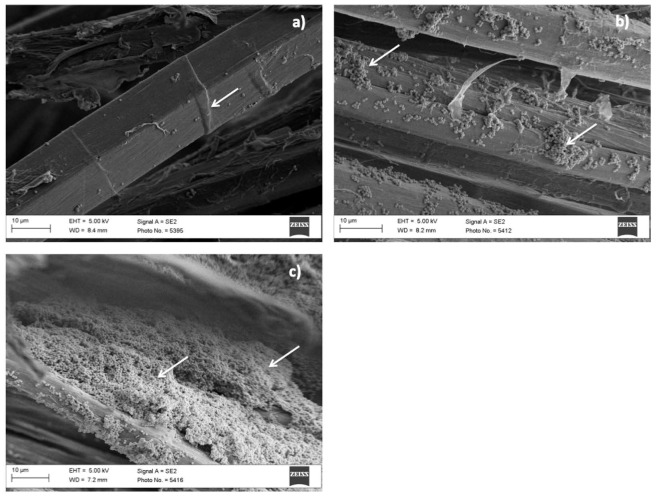
SEM pictures reporting different stages of bacterial adhesion and biofilm formation on flax fibers. Single cells of *S. aureus* adhered onto the substrate (**a**); initial steps of biofilm formation locally aggregated onto the surface of the fiber (**b**); dense structured biofilm completely covering the flax substrate (**c**).

In their study, Thuptimdang et al. analyzed the effect of silver nanoparticles on *Pseudomonas putida* biofilms at different levels of maturity and showed a higher effect of AgNPs on less mature biofilms [86]. Paladini et al. demonstrated the efficacy of silver nanocoatings deposited on textile materials for wound dressing in preventing bacterial adhesion and biofilm formation [87,88,89]. Figure 2 reports the scanning electron microscopy images of *S. aureus* colonies adhered and proliferated on flax fibers and different stages of bacterial proliferation and biofilm formation, from single bacterial cells (Figure 2a) to a dense and thick layer of bacteria (Figure 2c).

## 4. The Role of Silver in Wound Healing

The use of silver in wound management is very ancient. In Egypt, in 1850 BC, silver was applied to wounds; in addition, the textbooks by Hippocrates described the positive effects of silver in wound healing [90]. Today, due to their broad-spectrum antibacterial capability, silver-based creams and ointments, as well as AgNPs-based biomedical products, such as wound dressings, are commercially available for different medical applications [91].

Due to the attack of infectious diseases and the development of antibiotic resistance, pharmaceutical companies and scientists are looking for novel antibacterials [92]. Indeed, the scientific interest in silver nanoparticles and biopolymers for wound healing applications significantly increased in last years, as demonstrated by the Scopus publication history during the period 2013–2017 based on the keywords “silver nanoparticles”, “silver nanoparticles-biomaterial” and “silver nanoparticles-wound healing”, which generated 25, 593, 316, and 273 documents, respectively [91]. Sim et al. reviewed the patented silver-based products in the decade 2007 to 2017, including the use of antimicrobial silver for clinical and medical use, for personal care, for domestic, agricultural, and industrial applications. In 10 years, about 5000 new applications have been registered, mainly from Asian countries and in the Chinese language with 20% of patents in English [93]. Thus, over the last years, the interest in antimicrobial silver increased significantly, as demonstrated by the increased number of publications and patents. In 2000, the number of patented applications based on antibacterial silver was less than two hundred, while in 2017, it was almost 1500 [94].

In wound dressing applications, silver-based products have been patented and commercialized with more complex designs and improved efficacy compared to standard dressings [93].

Biopolymers combined with a bioactive antimicrobial, antibacterial, and anti-inflammatory nanoparticles have great potential in wound care to promote wound healing [95], particularly in the management of diabetic foot ulcers (DFUs), which still represent an enormous issue and are related to high amputation rates and clinical costs [95]. Dai et al. developed an antimicrobial peptide-AgNPs composite and have tested its wound healing properties in vivo on a diabetic rat model, demonstrating improved wound healing without side effects on dermal tissues and enhanced interactions between peptide and lipopolysaccharide moieties on bacteria, thus also indicating a wide-spectrum activity without inducing bacterial resistance [96]. The multilevel antibacterial effect of silver considerably reduces the chances for microorganisms to develop resistance and increases the effectiveness against multi-drug-resistant organisms [92]. The wound healing property of AgNP was tested by Kumar et al. on wound models in albino rats. The animals were treated with cream formulations containing different percentages of AgNPs and, at the highest percentage of silver, they showed reduced wound area, increased collagen deposition, few macrophages, tissue edema, and more fibroblasts [91]. In BALB/C mice with a thermal injury, silver NPs (14 nm) coatings on bandages reduced inflammation and scarring, eliminated bacterial growth, and accelerated the healing [97]. Gong et al. also developed biosynthesized AgNP based ointments, demonstrating the efficacy of AgNPs in upgrading wound healing activity and improving wound contractions in albino rats [98]. The results obtained by Adibhesami et al. demonstrated in vivo the positive effect of AgNPs on mice wound inoculated with *Staphylococcus aureus*, the most common pathogen involved in wound infections. In this study, accelerated the treatment of infected wounds were observed without side effects, along with enhanced remodeled wound areas [99]. On streptozotocin-induced diabetic mice model, nanobiocomposites impregnated with silver nanoparticles tested by Singla et al. demonstrated accelerated wound healing through significantly increased expression of collagen and growth factors, improved re-epithelialization, vasculogenesis and collagen deposition compared to control groups [100].

Indeed, an active role in wound healing was attributed to silver, and, along with its distinctive role in preventing infection, silver nanoparticles can also drive the differentiation of fibroblasts into myofibroblasts, which in turn promotes the wound contraction, quickens the healing rate, and stimulates the proliferation and relocation of keratinocytes [92,95]. Liu et al. studied the effect of AgNPs on keratinocytes and fibroblasts in an excisional wound model in rodents. Their histological studies and ex vivo wound model experiments demonstrated that AgNPs improve the proliferation and migration of keratinocytes from the edge to the center of the wound center and trigger the differentiation and maturation of keratinocytes, thereby promoting wound contraction. The wound closure of the AgNP-treated group exhibited an accelerated rate [101,102]. The response of primary human keratinocytes and fibroblasts after incubation with AgNPs was studied by Frankova et al., which compared the influence of AgNPs on the most exposed cell lines during the wound-healing process. The authors suggested that AgNPs can support the wound-healing process through both bacterial growth inhibition and the production of proinflammatory cytokines [103]. You et al. investigated in vitro and in vivo the effect of nanosilver on wound healing. They found that, at a concentration of 10 ppm, silver nanoparticles promoted the migration of fibroblasts, which also expressed higher levels of the marker α-smooth muscle actin (α-SMA), thus indicating the capability of silver to transform fibroblasts into myofibroblasts and to speed the healing process. The authors concluded that silver nanoparticles with proper size and concentration could represent a valuable tool to maintain a reasonable activation of macrophages, modulating the local inflammatory response [104]. The inflammatory response associated with the topical delivery of silver nanoparticles was investigated by Tian et al. in a mouse model. In particular, due to the important role of cytokines in wound healing, the authors analyzed the expression patterns of IL-6, TGF-β1, IL-10, VEGF, and IFN-γ by using a quantitative real-time RT-PCR. They found differences in mRNA levels of various cytokines, confirming modulated cytokine profile by silver nanoparticles. The authors also demonstrated reduced scar appearance in the presence of nanosilver and supported the beneficial application of AgNPs in wound care [105].

The study performed on AgNPs containing dressings by Velázquez-Velázqueza et al. demonstrated anti-biofilm activity against *P. aeruginosa* and compatibility to human fibroblasts. The authors associated the anti-biofilm capability of AgNPs (metallic and ions) to a potential interference, inhibition, and/or regulation of the exopolysaccharides (EPS) produced by bacteria [61]. 

Prevention from microbial accumulation plays an important role in rapid wound management and to avoid high costs of antibiotic use [52]. Controlling the bacterial burden in a wound is crucial for successful wound repair and, in vivo, a reduction of the bacterial burden by silver-containing dressings was demonstrated, accelerating the wound healing progress [25]. 

Rath et al. described the use of a collagen nanomatrix incorporating a reservoir of AgNPs for accelerated wound healing. In vivo studies demonstrated good wound-healing properties due to intrinsic antibacterial, anti-inflammatory, and hemostatic properties [106]. Metcalf et al. investigated the application of silver-based antimicrobial dressings on 112 difficult-to-heal wounds and, in 83% cases, a progression in management of exudate, biofilm, and wound healing was observed [107].

A wide range of products can be functionalized with silver nanoparticles, such as bandages, gauzes, sutures, plasters, and also many other creams and ointments can incorporate AgNPs for wound healing application [108]. Along with antimicrobial properties, silver treated textile materials and surgical sutures demonstrated in vitro improved wound healing properties, thus also indicating a positive effect of silver in terms of cell migration and proliferation [73,74,88]. For further improved wound healing, a synergistic effect of silver and sericin was demonstrated for wound healing, suggesting a successful combination between silver and silk proteins for improved antibacterial properties and tissue regeneration and opening new options for the development of completely natural wound dressing biomaterials [75].

## 5. Conclusions and Future Trends

Antimicrobial resistance is a growing global concern, which involves wound care and issues related to the nature of the wound and the wound care [109]. Infections causing non-healing wounds still remain a serious challenge, and clinical data indicate the presence of biofilm and its relation with wound chronicity [23,110].

Although many efforts have been made for designing new antimicrobial agents, wound management still represents a serious clinical issue and, today, it also represents an intense area of research [39,110]. Due to the costs and the complexity of treating chronic wound infections associated with biofilms, new and alternative strategies for effective anti-biofilm treatment are necessary and, in this scenario, nanotechnology offers novel approaches for improved wound healing, for the development of a class of nanoparticles with unique properties [39,110,111], and for new therapeutic options for dermatological infectious and skin diseases [112].

Recent findings allow for the production of wound dressings for the delivery of active molecules and/or drugs to the wound site [23]. A significant amount of research data has provided evidence about the beneficial effects of silver nanoparticles in biocompatible and nanostructured materials and devices [51]. However, the mechanism of interaction between silver nanoparticles and bacteria, along with clinical and toxicity studies, still requires intense research [112].

The VULCAN study is, for example, a multicenter, prospective randomized controlled trial performed on 213 patients with venous leg ulceration to evaluate the effectiveness of silver dressings in comparison with non-antimicrobial non-adherent dressings. The choice of the dressing was made by the clinician and the complete ulcer healing, time to healing, quality of life, and cost/effectiveness ratio were evaluated. The study was concluded assessing that no significant differences were observed between the use of silver dressings and non-antimicrobial non-adherent dressings, thus suggesting that no advantage is associated with the use of silver-donating dressings [113]. Leaper and Drake extensively described some limitations of this study and evidenced that, due to the intricate nature of the wound healing process, the choice of the dressing depends on many parameters, such as type and location of the wound, geographical area of the patient, and patient preference. Most importantly, in the VULCAN study, the researchers only considered the venous ulcer as the type of chronic wound, using silver dressings on non-infected wounds for long periods, thus not satisfying the current recommended best practice [114,115] that addresses the use of silver dressings exclusively in the case of clinical signs of infection or in patients at high risk of infection [115]. Moreover, although Cochrane reviews concluded that the use of silver dressings for promoting wound healing and preventing infections is not supported by sufficient evidence, 26 random controlled trials (RCTs) incorporated 2,066 patients with positive results and a number of clinical and case studies found that silver dressings can promote wound healing and prevent wound contamination [114,115,116]. A group of experts evaluated the clinical studies on silver in wound management published during the period 2000–2015, identifying 851 articles, of which 173 were included and categorized. The meta-analysis showed evidence supporting the use of silver in wound management for limited periods, with advantages in terms of antimicrobial effects, improved quality of life, and good cost-effectiveness [90]. It is evident that all silver dressings are not equally effective and that more clinical studies are necessary. However, the available data demonstrated the big potential of silver in wound management [115]. PubMed, EMBASE, Cochrane and other databases report relevant randomized controlled trials, in which nanocrystalline silver dressings results were more effective than silver sulfadiazine [SSD] and silver nitrate in reducing hospitalization, pain, and infection rates in superficial and deep partial-thickness burns [117].

Other in vivo studies by Mohseni et al. compared antimicrobial dressings conjugated with silver sulfadiazine (SSD) and silver nanoparticles (AgNPs) for chronic wound healing. Although at similar concentration SSD and AgNPs demonstrated strong and equal anti-microbial activity against *S. aureus,* AgNPs demonstrated higher biocompatibility, faster healing rate, epithelization, and dermal regeneration. The authors concluded that nano-silver was more efficient and more promising than silver sulfadiazine in wound healing application [118].

Tian et al. tested silver nanoparticles and SSD in a thermal injury model comparing the rate of healing of deep partial-thickness wounds. They found less hypertrophic scarring and nearly normal hair growth on the wound surface treated with silver nanoparticles and confirmed the accelerated wound healing by using silver nanoparticle [105]. Clinically, there is no reliable evidence to date about treatment failures related to silver resistance, and silver treatment results show silver treatment is still effective even when wound bacteria have silver-resistant genes [110].

In low concentrations, silver has been indicated as non-toxic material to humans, and it has been assessed as a promising material in pharmaceutical and biomedical fields [119,120].

Although silver nanoparticles have been investigated for their superior physical, chemical, and biological properties, some issues related to synthesis methods, potential risks to health and the environment and scale-up production still require future works to promote a safer and more efficient use of the nanoparticles [121].

The application for wound dressing biomaterials, in particular, requires intense multidisciplinary research involving researcher, biotechnologists, and clinicians. Engagement, alignment, and collaboration between wound care professionals and related teams and governments on antimicrobial stewardship will help to generate further progress in the fight against antimicrobial-resistant infections in wound care [109].
